# Monitoring warning criterion of acoustic emission active waveguide system based on loess deformation and failure

**DOI:** 10.1038/s41598-024-62030-1

**Published:** 2024-05-18

**Authors:** Ke Zhang, Liang Wang, Guoqiang Meng

**Affiliations:** 1 Department of Earth Sciences and Engineering, Taiyuan University of Technology, Taiyuan, 030024 China; 2 Shanxi Low Carbon Environmental Protection Industry Group Co., Ltd., Taiyuan, 030001 China; 3 Shanxi Construction Engineering Group Co., Ltd., Taiyuan, 030001 China

**Keywords:** Geological disaster, Monitoring and early warning, Active waveguide system, Acoustic emission criterion, Environmental sciences, Natural hazards

## Abstract

The construction of acoustic emission criterion system is crucial for monitoring and providing early warning of geological hazards. In the current soil acoustic emission monitoring methods, the signal generated by soil deformation and failure is weak and experiences high attenuation, resulting in a low level of the monitored signal. One approach to enhance the quality of monitoring data is by utilizing the active waveguide model. However, the current research on the active waveguide model system is not extensive. To address these issues, a set of active waveguide system was designed to improve the data quality of acoustic emission monitoring and early warning. The deformation and failure process of loess monitored by acoustic emission was divided into stages, and the precursor information of acoustic emission for geological disasters in loess areas was comprehensively deconstructed. The data quality advantage of the active waveguide model was verified through comparative experiments of with the passive waveguide model. This study investigates the AE signal characteristics of the active waveguide model. It explores various aspects such as the AE waveform parameter characteristics, the discrimination method for failure mode based on RA–AF value, the AE r-value characteristics, the AE b-value characteristics, and the frequency-amplitude characteristics. The study reveals the evolution law of AE signals in the active waveguide model, including early warning signs and failure morphological characteristics. Furthermore, it constructs a warning criterion for the active waveguide system. The development of this criterion system is of great importance in guiding the monitoring and early warning of geological disasters in loess areas.

## Introduction

Geological hazards, as destructive geological events, pose a significant threat to human life, property, and the overall living environment^1,2^ . For instance, severe landslides and collapses have been observed in countries like Germany, Kyrgyzstan, and China^3–12^. In the loess area, often referred to as the ‘thousands of gullies’, the presence of numerous gullies and broken landforms has created an extremely fragile geological environment, leading to the extensive occurrence of geological disasters^13,14^ . Landslides occur due to the unique volumetric characteristics of loess, which can behave as a weakly cemented mass moving along interconnected joints, like the failure mechanism of rock slopes^15^ . The thickness and loose nature of loess make it susceptible to collapse, rapid disintegration, and gully erosion^16,17^ . Previous monitoring methods for geological disasters have primarily focused on the stress and strain of soil^18–20^ . However, these methods are not effective in timely obtaining failure precursor information for sudden geological disasters in loess areas.

Acoustic emission (AE) monitoring tool is a crucial tool to realize the short-term and imminent warning of loess slope deformation and failure disasters. It effectively captures the characteristics of structural instability and collapse of loess slope by using the characteristics of AE signals. The AE signal comes from the stress wave generated by the sudden release of stored strain energy in a local area of the material, which has a large amplitude, a fast speed, and a high frequency^21,22^ . Local deformation and failure of soil occur under the disturbance of surrounding environment, and energy release occurs due to the fracture of cement, extrusion, and friction between particles in soil. And dissipated as elastic or acoustic waves^23–26^ . Soil deformation or failure can generate significant AE in the frequency range of 10 ~ 100 kHz^27,28^ . Nevertheless, the AE energy in soil is too low and the attenuation is too fast, and the attenuation range in soil is more than 10 dB/cm^29^ . To avoid the energy attenuation of the AE wave, an active waveguide model was proposed, in which a waveguide rod was buried in the soil, the AE waveguide was guided to the sensor located on the ground, and a filling material was filled around the waveguide rod^30^ . The difference between the active waveguide model and the passive waveguide model is that the AE source received by the sensor is different, and the active waveguide model generates AE signals from the filling material^31–35^ . The passive waveguide model generates AE signals from soil failure^36^ .

In the AE signal analysis method, the RA-AF value can be used to identify the failure mode. The RA value is defined as the ratio of the rise time to the maximum amplitude, and the AF value is defined as the ratio of the ring-down counts (RDC) to the duration^37,38^ . The concept of AE b-value was first proposed in seismology. Scholars described in detail the evolution law that the seismic frequency becomes weaker with the decrease of magnitude, and then proposed the b-value calculation formula^39^ . Now more and more experts apply the b-value of AE to geotechnical engineering and have achieved fruitful results^40^ . Uniaxial compression tests were carried out to investigate the b-value characteristics and the denoised AE signal’s spectral multifractal features are analyzed^41,42^ . The characteristics of AE b-value and material fracture evolution of backfills with different ratios of cement and tailings under compression were studied through uniaxial compression AE tests^43^ . The results show that the characteristics of AE b-value and frequency-amplitude can be used to obtain the precursor warning information of internal crack development in the process of structural deformation and failure.

Nevertheless, the research on the active waveguide model is still in its infancy, especially there is no direct research on the monitoring and early warning criteria of the AE active waveguide model based on geological disasters in loess areas. Therefore, it is urgent to carry out the simulation test of active waveguide model for monitoring and early warning of loess deformation and failure, to explore the evolution law of AE signals in the process of active waveguide model monitoring, and to realize the construction of disaster early warning criterion system in loess areas.

In this study, the active waveguide model is taken as the key research object, and the compression loading test of the active and passive waveguide models is carried out. Through the comparative analysis of the test data of AE waveform parameters in the monitoring process, the data quality advantage verification of the active waveguide model is realized. Based on the method of RA-AF value failure mode discrimination, the failure mode of loess slope monitored by active waveguide model is discriminated, and the characteristics of AE b-value and various waveform parameters of the active waveguide model are revealed, and the process of loess deformation and failure is divided into stages according to the time distribution law of b-value and the characteristics of AE waveform parameters. At the same time, the characteristics of AE r-value and frequency-amplitude are explored, and the monitoring and early warning criterion of active waveguide system is constructed by combining the characteristics of AE signals.

## Experimental methods

### Active waveguide modeling transmission mechanisms

**Figure 1:**
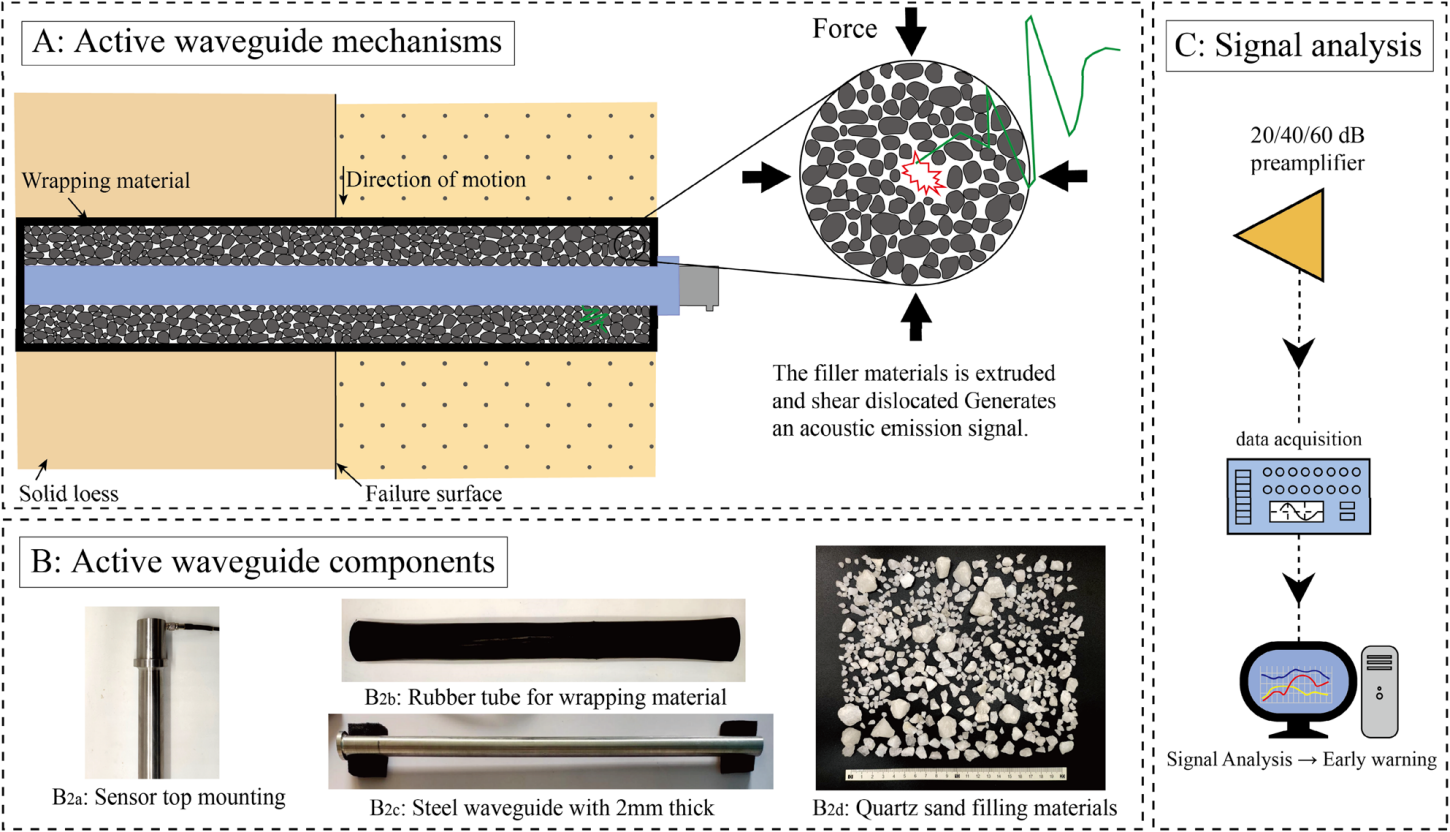
Schematic diagram of the AE transmission mechanism of the waveguide model. (**A**) Active waveguide mechanisms. (**B**) Active waveguide components. (**C**) Signal analysis.

The active waveguide model is composed of an internal steel waveguide rod and an external rubber tube, the gap between the inner and outer tubes is filled with filling materials, and the upper and lower ends of the active waveguide model are sealed to prevent the loss of filling particles and the inflow of surrounding geological particles. The active waveguide model is installed in the borehole of loess slope, and the waveguide model is mainly subjected to compression and shear. The AE source of the active waveguide model is generated by the filling material, the loess deformation produces extrusion and friction on the filling material, and the AE signal is generated by the compression and shear dislocation of the filling material and the collision with the waveguide rod. The schematic diagram of the waveguide model is shown in Fig. 1. The active waveguide model consists of the sensor installation method, filling material, waveguide rod and wrapping material. When relative displacement occurs in the process of loess instability and failure, the active wave-guide model experiences shear forces, with its shear surface located at the vertical section of the active waveguide model.

In the passive waveguide model, the sensor is directly connected to the steel pipe and then embedded in the loess. The AE signal generated by the weak deformation of the loess is so small that it is difficult to be effectively monitored. The active waveguide model can detect significant AE signals in the face of slight loess deformation, so the active waveguide model has high sensitivity for the deformation and failure monitoring of loess slopes. At the same time, the greater the displacement rate of loess is, the greater the intensity of compression and shear dislocation between the particles of the filling material in the active waveguide model is, and then more AE signals with higher quality of monitoring data are released. The AE active waveguide model can achieve the purpose of obtaining the precursor information of loess slope deformation and failure in time. AE signals are generated by the filling material at and near the failure surface and are transmitted along the steel waveguide rod to the sensor at the top. The main function of the active waveguide model is real-time monitoring, quantification and early warning of loess slope deformation and failure, which is helpful to prevent geological disasters in loess areas in time and achieve the purpose of high-precision and high-quality real-time monitoring and early warning of loess slope instability and failure.

### Experimental design

Landslide and collapse are the most common and serious geological disasters in the Loess Plateau. The development of vertical joints and toe erosion are the key reasons for the occurrence of geological disasters in loess areas^44^ . There are many vertical slopes in the Loess Plateau area, and the linear slope accounts for the largest proportion of geological disasters in the loess area of Shanxi Province. The loess used in this test is taken from Chengzhuang Town, Linxian County, Shanxi Province, which belongs to Q_3_ Malan loess. The linear slope is selected as the simulation research object, and the filter method is used to remove the impurities such as sand and plant roots in the loess. The test model size is set as 300 mm × 100 mm × 100 mm, and the model box is made of acrylic plexiglass, which is convenient for real-time observation of the deformation and failure of loess during the test.

The preparation process of the loess body used in the model test includes loess sample preparation, drying by a dryer, loess crushing by a crusher, water content preparation, layer-by-layer compaction, etc. According to the actual measurement, the dry density of loess in Linxian County is about 1.42 g/cm^3^, and the remolded loess with dry density of 1.42 g/cm^3^ is prepared, the water content is 10%, and the density is 1.4626 g/cm^3^.

**Figure 2:**
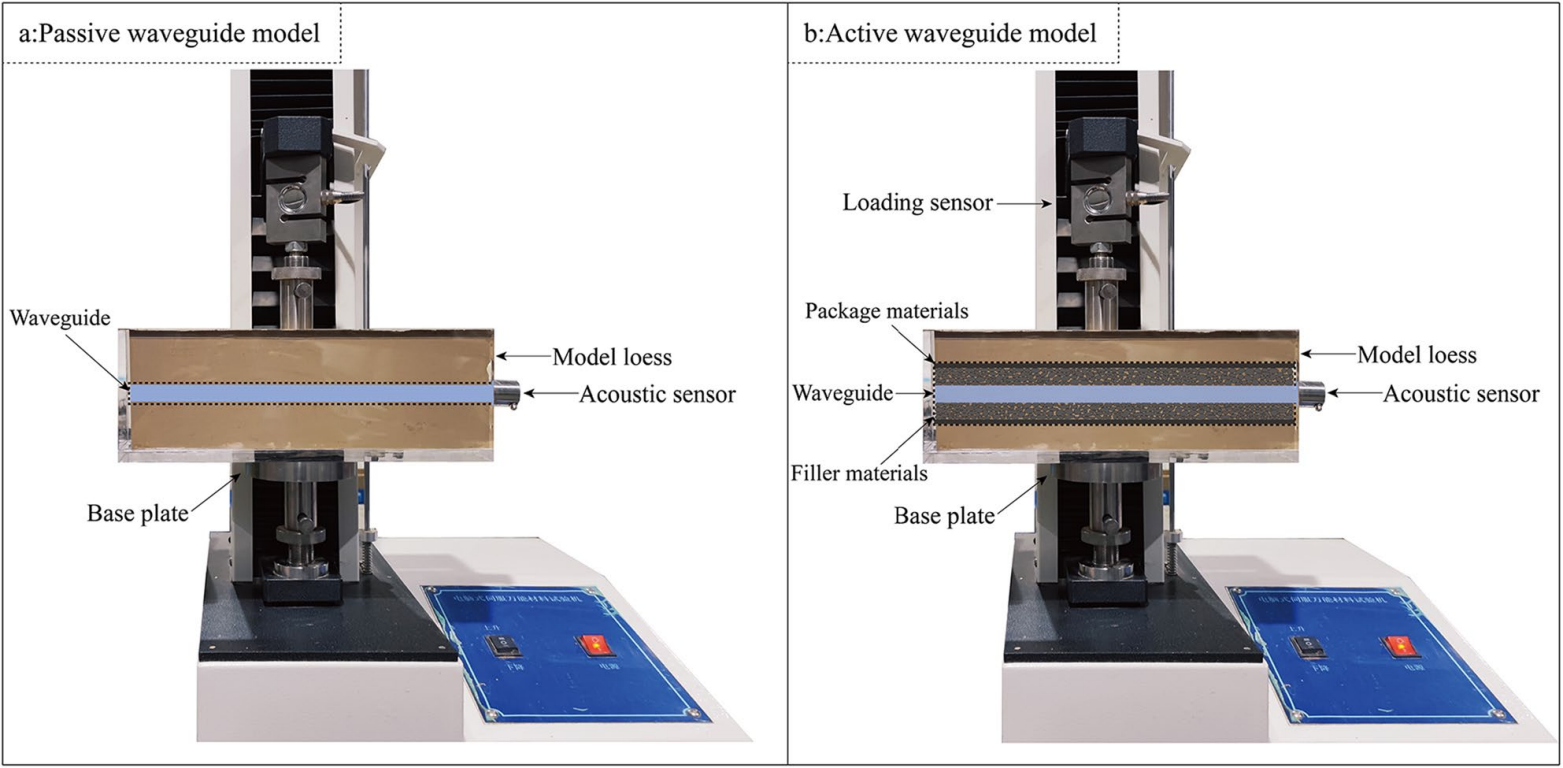
Waveguide model test set. (**a**) Passive waveguide model. (**b**) Active waveguide model.

The AE test devices of the active waveguide model and the passive waveguide model are shown in Fig. 2. The AE test system comprises a compression loading instrument and an AE monitoring and analyzing device, wherein the compression loading instrument adopts a computer servo universal material testing machine, and the AE instrument adopts a DS5-8B AE instrument. The length of the steel waveguide rod is 600 mm, and the sensor is installed on the top of the waveguide rod. The compression loading tests were carried out on the active waveguide model and the passive waveguide model, respectively, with the displacement rate controlled at 1 mm/min and the test duration of 10 min. Active waveguide model configuration: 2 mm wall thickness steel waveguide rod, filling material is quartz sand with particle size range of 3 to 20 mm, outer wrapping material is 1 mm wall thickness rubber tube, and both ends of the wrapping material are sealed to prevent gravel loss. Passive waveguide model configuration: 2 mm wall thickness steel waveguide rod, no filling material, and no outer wrapping material. Inserted into the loess, through the preset failure surface, and placed on the servo universal testing machine to carry out compression loading test.

## Results and analysis

### Waveform parameter results

**Figure 3:**
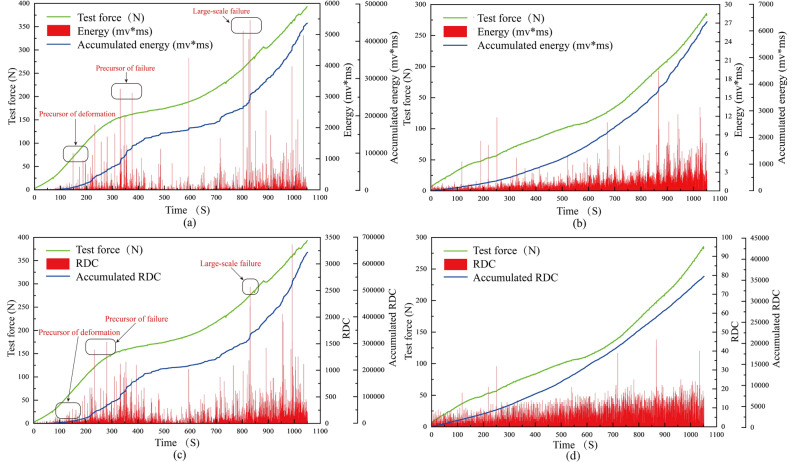
Test force vs. AE energy parameters. (**a**) Active waveguide model energy. (**b**) Passive waveguide model energy. (**c**) Active waveguide model RDC. (**d**) Passive waveguide model RDC.

Among the respective AE waveform parameters, the significant waveform parameter with crucial influences for quantifying deformation was obtained through research. Moreover, the trends of waveform parameters, mechanical parameters, and deformation process with time as well as the correspondence between them were obtained. The AE waveform parameter signals and stress test results obtained in this study are shown in Fig. 3. The energy parameter of the active waveguide model reaches a maximum of 5462.06 mV × ms at the 830.64 s/14.06 mm displacement, and the cumulative energy is 446,936 mV × ms. The passive waveguide model energy parameter reaches a maximum value of 19.34 mV × ms at the 867.20 s/14.68 mm displacement, and the cumulative energy is 6338 mV × ms. The RDC of the active waveguide model reaches a maximum of 3365 at the 991.85 s/16.79 mm displacement, and the cumulative RDC is 643800; the RDC of the passive waveguide model reaches a maximum of 46 at the 867.20 s/14.68 mm displacement, and the cumulative RDC is 35758. It is found that the waveform parameters (energy and RDC) of the active waveguide model are much larger than those of the passive waveguide model.

**Figure 4:**
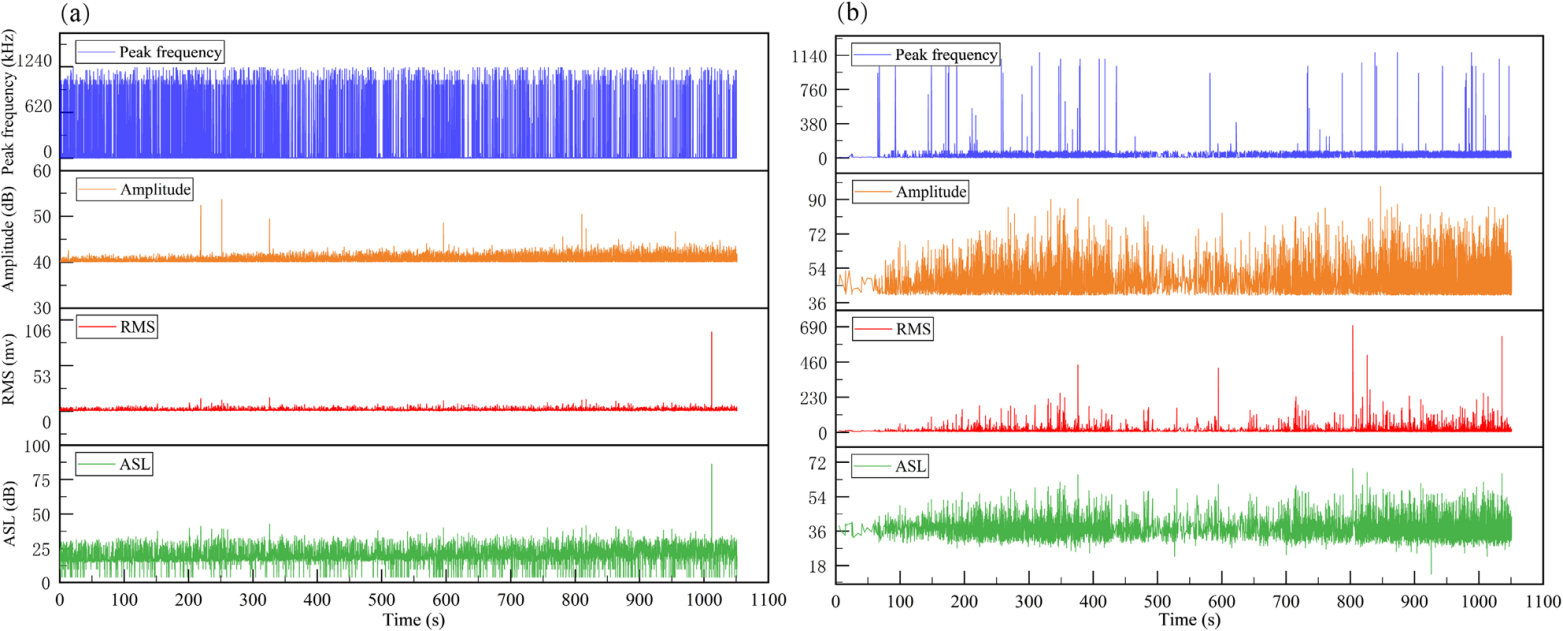
Average signal level, root mean square, amplitude and peak frequency plotted against time for waveguide. (**a**) Passive waveguide model. (**b**) Active waveguide model.

The average signal level (ASL), root mean square (RMS), amplitude, and peak frequency characteristics over time for the AE active and passive waveguide models from this study are shown in Fig. 4. The comparison shows that the RMS and Amplitude of the active waveguide model are much higher than those of the passive waveguide model, the ASL is slightly higher than that of the passive waveguide model, and the Peak frequency is much lower. Therefore, from the overall distribution, the signal characteristics of the passive waveguide model are high frequency and low amplitude, while the signal characteristics of the active waveguide model are low frequency and high amplitude, which follow the distribution law of weak sound signal and strong sound signal.

**Figure 5:**
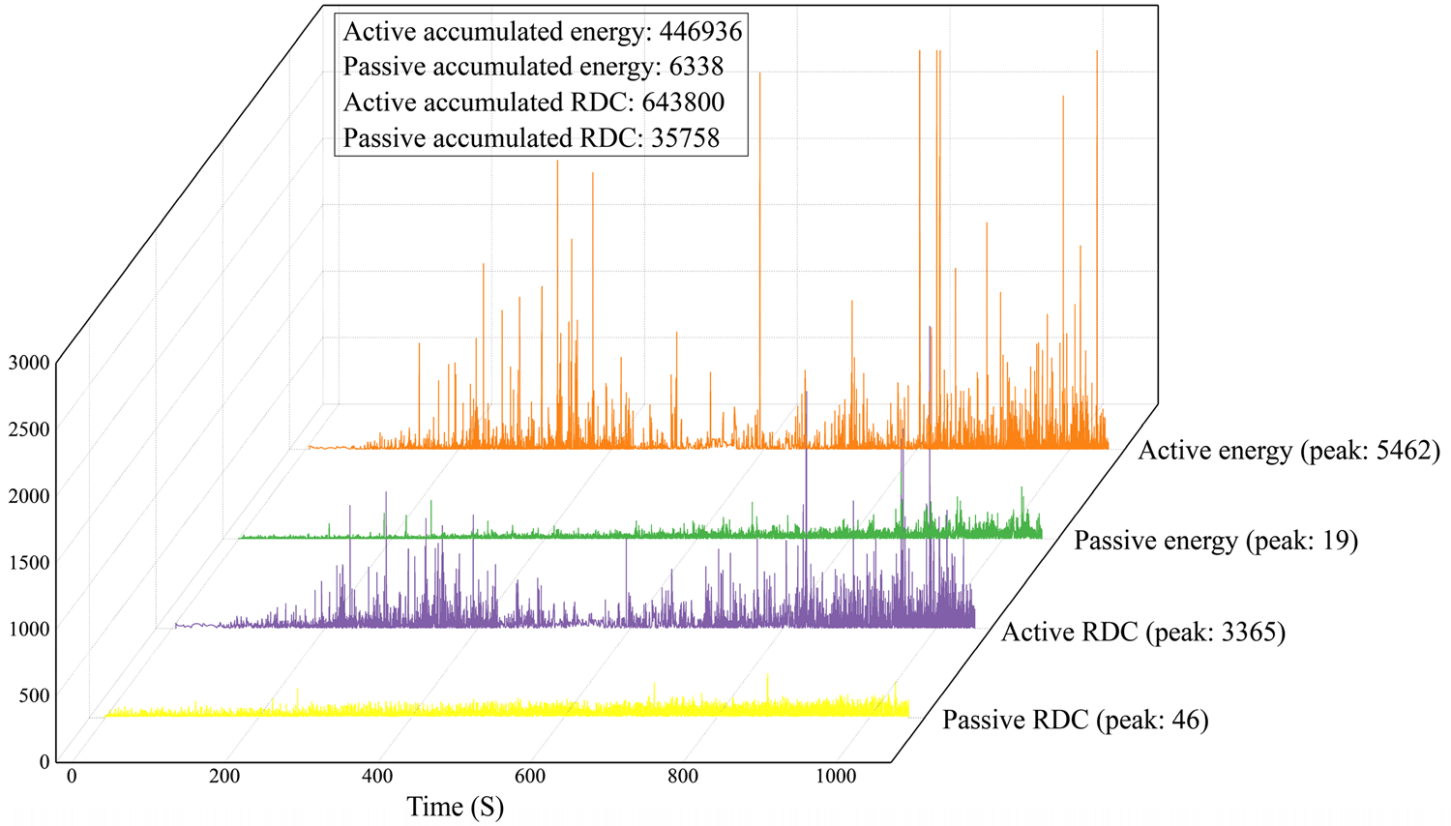
Comparison and analysis of waveform parameter characteristics of active waveguide model and passive waveguide model.

The waveform parameter characteristics of the active waveguide model and the passive waveguide model are compared and analyzed as shown in Fig. 5. The active waveguide model generates 11,329 AE energy parameters, and the passive waveguide model generates 9104 AE energy parameters. In the active waveguide model, there are 3228 AE data with energy greater than 20 mV × ms, and 8102 AE data with energy less than 20 mV × ms. In the passive waveguide model, there are 9104 AE data whose energy parameters are all less than 20 mV × ms. For the active waveguide model, there are 3260 RDC greater than 50, and 8069 RDC less than 50. For the passive waveguide model, there are 9104 RDC less than 50. Therefore, the energy parameter greater than 20 mV × ms and the RDC greater than 50 in the AE data can be judged as the shear dislocation of the filling material.

From the comparative analysis of AE waveform parameter results, it is concluded that the AE waveform parameter data quality of the active waveguide model is significantly better than that of the passive waveguide model when monitoring the deformation and failure of loess slope. Thus, the active waveguide model proves to be superior in terms of data quality for monitoring and warning the deformation and failure of loess slope.

### Failure mode determination

The RA and AF values of AE are two important parameters for signal analysis and processing in order to characterize the internal failure mode of a structure. Research has demonstrated that when the AE RA-AF value signal exhibits the characteristic of shear wave as 'high RA value and low AF value', it indicates shearing failure. Conversely, when the signal shows 'high AF value and low RA value', it indicates tensile failure^45,46^ . Relative to tensile cracks, they have rapid energy release, shorter duration and rise time, and higher amplitude, hence "high AF and low RA values". Relative to shear cracks, which have slower energy release, longer duration and rise time, and lower amplitude, hence "high RA value, low AF value"^47^ . The diagonal line of the RA-AF value distribution diagram is taken as the dividing line between the tensile crack and the shear crack, the upper area of the diagonal line is the tensile crack, and the lower area of the diagonal line is the shear crack^48^ .

**Figure 6:**
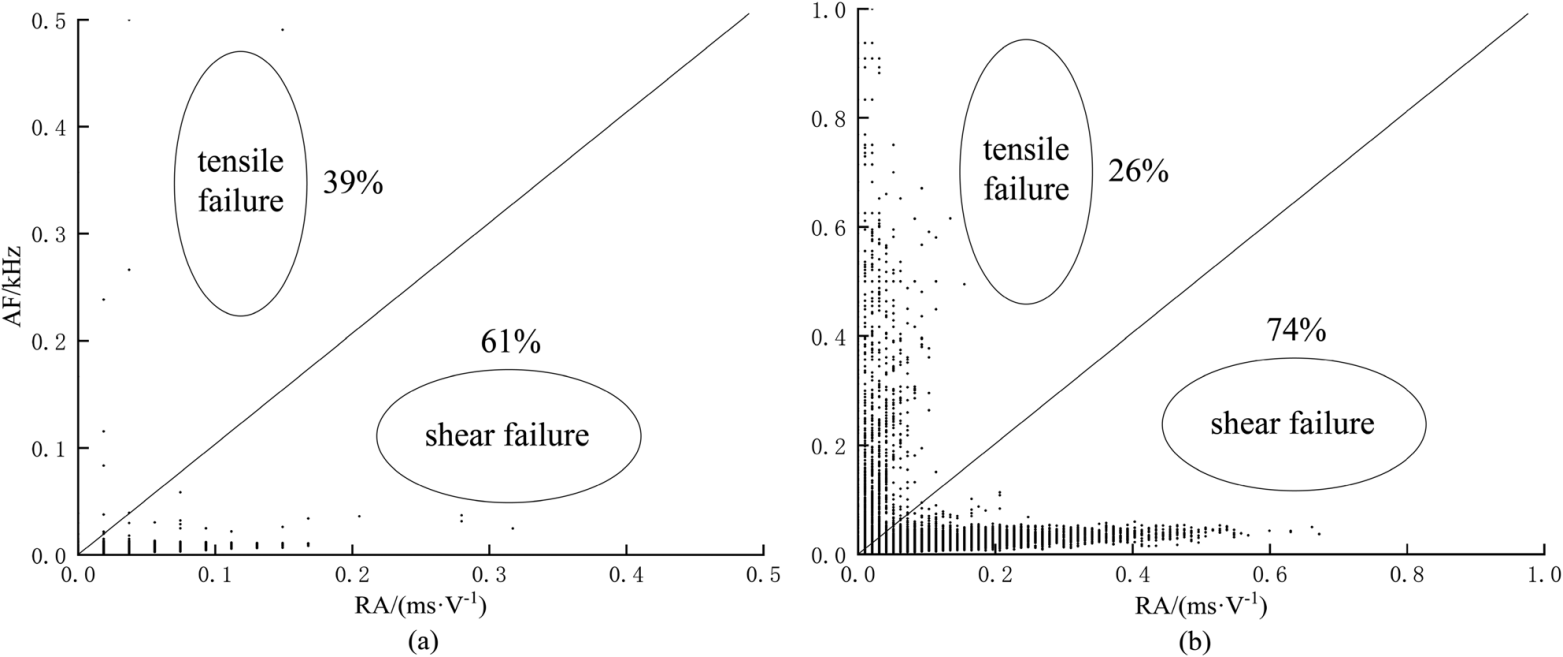
Discrimination of failure modes based on RA-AF values. (**a**) Passive waveguide model. (**b**) Active waveguide model.

The judgment results are shown in Fig. 6. The number of high RA values of the passive waveguide model is 5553, the number of high AF values is 3587, the proportion of high RA values is about 61%, the proportion of high AF values is about 39%, and the failure mode is shearing failure. For the active waveguide model, the number of points with high RA value is 8419, the number of points with high AF value is 2910, the proportion of high RA value is about 74%, and the proportion of high AF value is about 26%. In the compression loading test for monitoring loess deformation and failure in the active waveguide model, the failure mode is shearing failure, and the proportion of shear failure in the active guide model is larger than that in the passive guide model. This analysis method effectively determines the distribution of different cracks in the process of failure, which can provide an important theoretical basis for the identification and acquisition of loess deformation and failure precursor information.

### AE r-value characteristics

As one of the methods for analyzing AE signals, the AE r-value is defined as the ratio of the impact number to the cumulative energy^49,50^ . The r-value of AE is inversely proportional to the cumulative energy of a single waveform signal. A smaller r-value indicates a larger cumulative energy, suggesting a higher concentration of energy and a higher possibility of large-scale damage. This characterization can be used to assess the degree of energy concentration in the loading process of loess. The continuous decrease of the AE r-value of the active waveguide model reflects that the AE energy is higher, the number is less, and the active waveguide model is in a large-scale rupture state.

**Figure 7:**
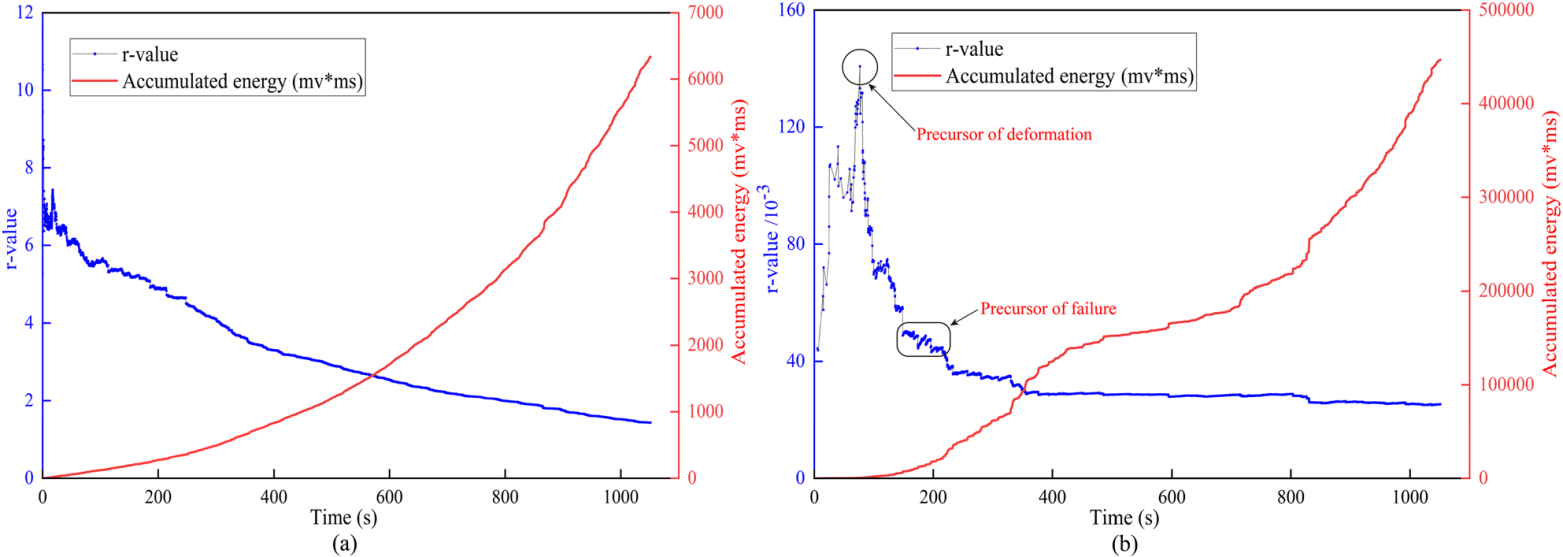
AE r-value characterization. (**a**) Passive waveguide model. (**b**) Active waveguide model.

The evolution law of the AE r-value of the waveguide model during the loading process is shown in Fig. 7. It is difficult to obtain the precursor information of loess deformation and failure because of the continuous reduction of the passive waveguide model. At the beginning of the active waveguide model, the r-value increases rapidly, which indicates that the AE energy is generated during the pressurization process, but the value is less. After a period of loading, the r-value decreases rapidly, which indicates that the AE energy with medium and high values begins to be generated, and the small-scale fracture in the loess is gradually bred. With the continuous loading, the r-value enters a stable reduction stage, reflecting the generation of many high-value AE energy, and the loess fracture changes from small-scale fracture to large-scale fracture.

### AE b-value characteristics and phasing

**Figure 8:**
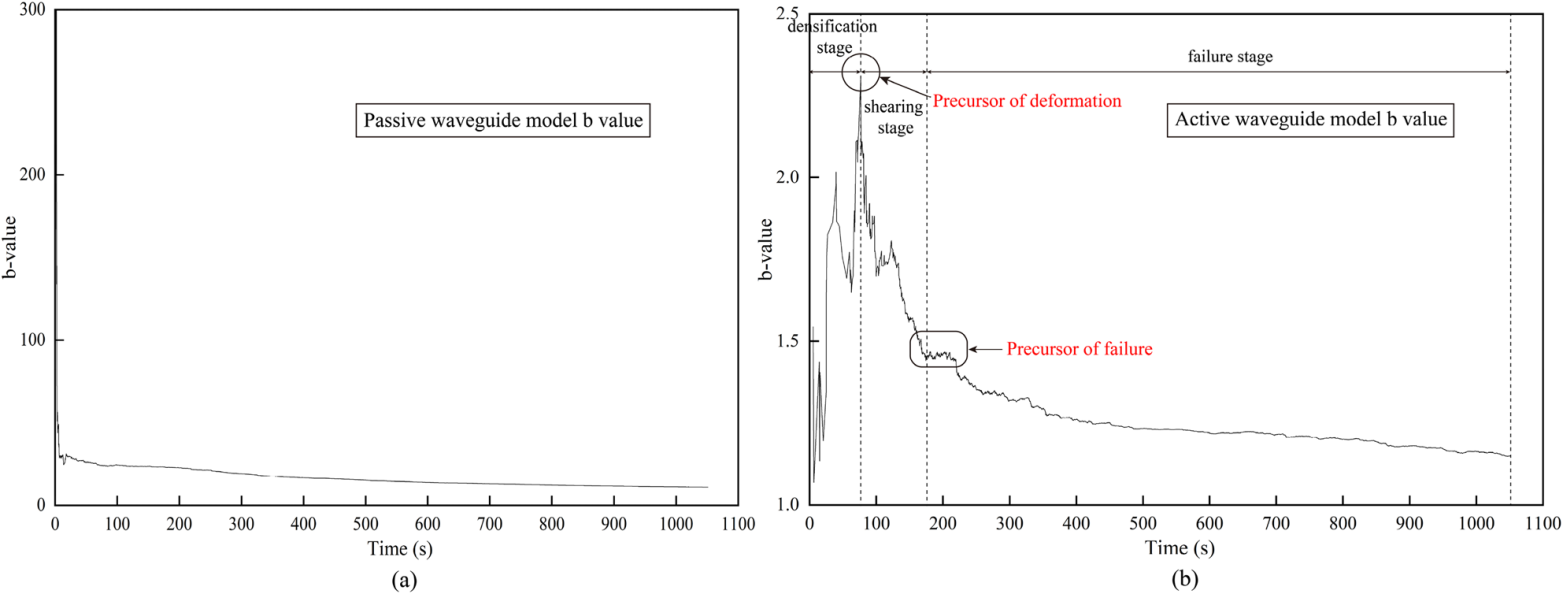
Acoustic b-value characterization. (**a**) Passive waveguide model. (**b**) Active waveguide model.

The maximum likelihood estimation method is commonly employed to calculate the AE b-value, which is used to assess the size of AE signal in rock and loess failure. The b-value represents the ratio of low amplitude events to high amplitude events in the AE signal. In this study, the AE b-value of the active waveguide model was calculated using the modified maximum likelihood method^51^ . The time distribution characteristics of the b-value in the waveguide model are depicted in Fig. 8.

In the passive waveguide model, the b-value continuously decreases throughout the loading process, except for a sudden increase at the beginning. This continuous decrease makes it challenging to effectively obtain warning precursor information. On the other hand, the AE b-value of the active waveguide model exhibits a sudden drop before shear deformation, followed by a stable reduction stage before failure. The deformation and failure monitoring of loess using the active waveguide model can be divided into three stages. Firstly, during the compaction stage (0–80 s), low amplitude AE signals appear, and the b-value shows an upward trend. Secondly, during the shear stage (elastic–plastic deformation stage) (80–180 s), high amplitude AE signals gradually emerge, and the b-value exhibits a rapid downward trend. Lastly, after 180 s, as the loess gradually deteriorates, numerous high amplitude AE signals appear, and the b-value shows a steady downward trend.

**Figure 9:**
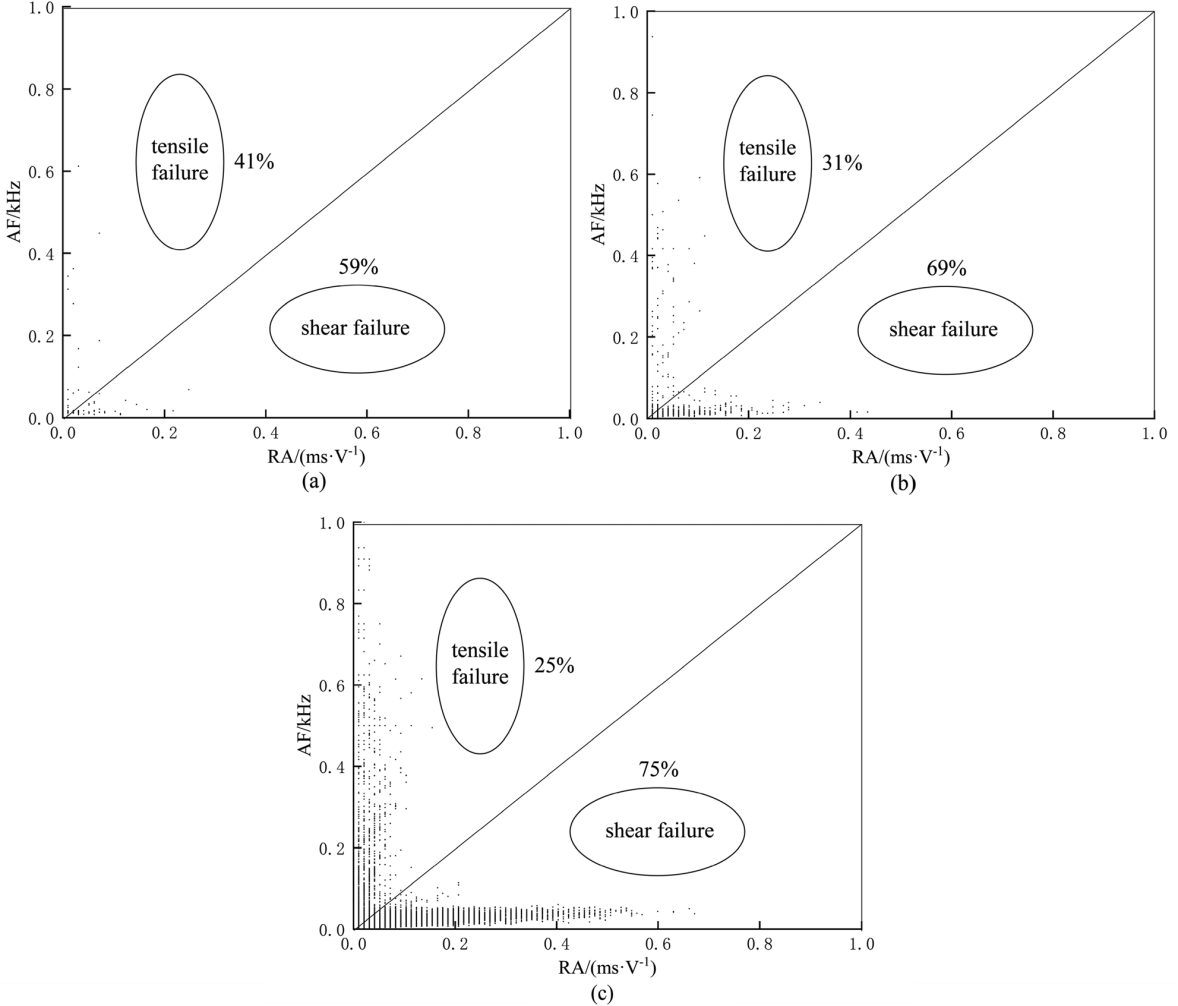
Percentage of different stages of failure modes based on RA-AF values. (**a**) Densification stage. (**b**) Shearing stage. (**c**) Failure stage.

After dividing the failure process of loess into three stages according to the AE b-value and the characteristics of AE waveform parameters, the proportions of tensile and shear failure modes in the three stages are also different based on the failure mode discrimination of RA-AF value, as shown in Fig. 9.Based on the characteristics of AE waveform parameters, the failure process discrimination can be determined using the RA-AF value and the AE b-value. Initially, during the loading stage, both the cumulative RDC curve and the AE RDC curve show a relatively flat trend, indicating a lower number of AE events and a stable distribution of micro fissures or pores in the loess, suggesting the compaction stage. However, as the loading continues, the RDC curve gradually rises, and the AE b-value curve starts to decline rapidly. This indicates an uneven distribution of shrinkage cracks and micro-pores in the loess, representing the shear stage (elastic–plastic deformation stage) and the gradual emergence of higher AE signals. Once the stress reaches 25% of the peak stress, the AE b-value steadily declines, indicating that the model loess enters the failure stage. At this point, there is a sharp increase in the number of AE events, a rapid increase in the proportion of large-scale failure, and the expansion and connection of cracks and pores in the loess. Ultimately, this leads to the formation of a fracture surface resulting in the failure of the model loess.

### AE frequency and amplitude characteristics

**Figure 10:**
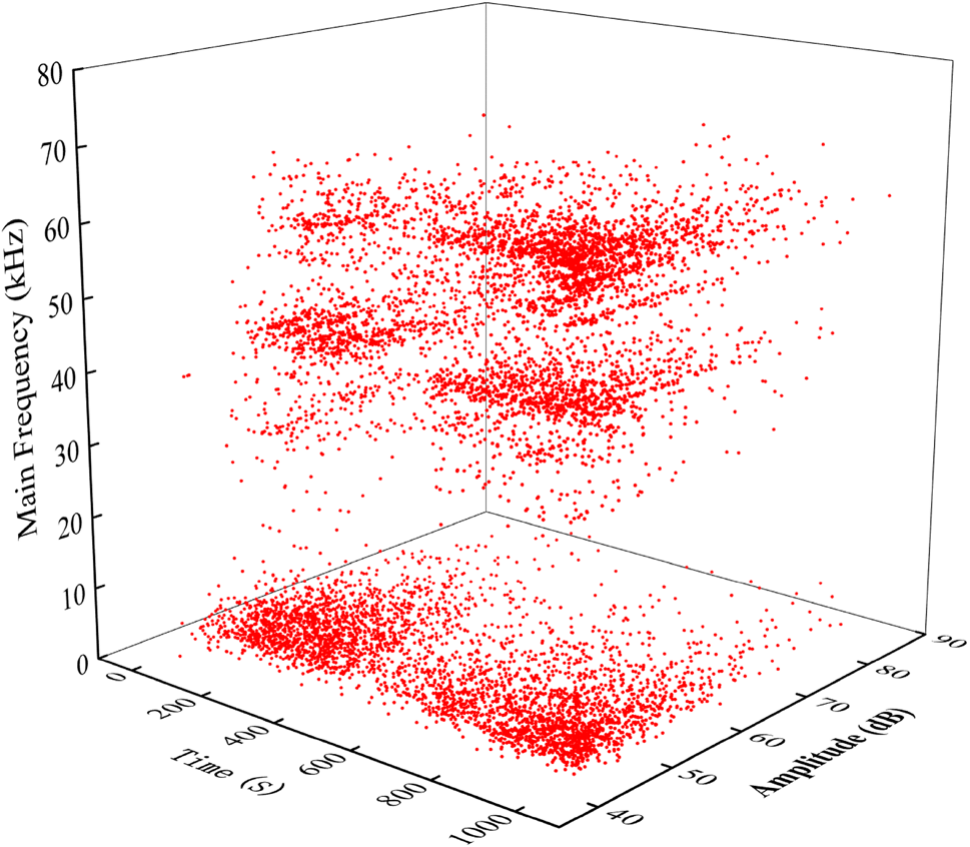
Active waveguide model time-major frequency-amplitude waveform parameters.

In the waveform parameters of AE signals, the characterization method also includes frequency and amplitude. The AE amplitude waveform parameter is an important characteristic parameter in AE signals, which is generally expressed by the decibel number corresponding to the output of 1 microvolt of the transmission element^52^ . Figure 10 shows the three-dimensional spatial waveform parameters of the active waveguide model in terms of time, amplitude, and dominant frequency. Upon analysis, it is observed that the amplitude level is low during the initial stage of low stress compaction. As the stress continues to increase and enters the shear stage, more high-amplitude signals start to appear. In the failure stage of the loess, a large number of high-amplitude signals are observed. The amplitude of AE signals can reflect the failure strength of loess, indicating that the deformation and failure of loess is a process that goes from small-scale failure to large-scale failure.

**Figure 11:**
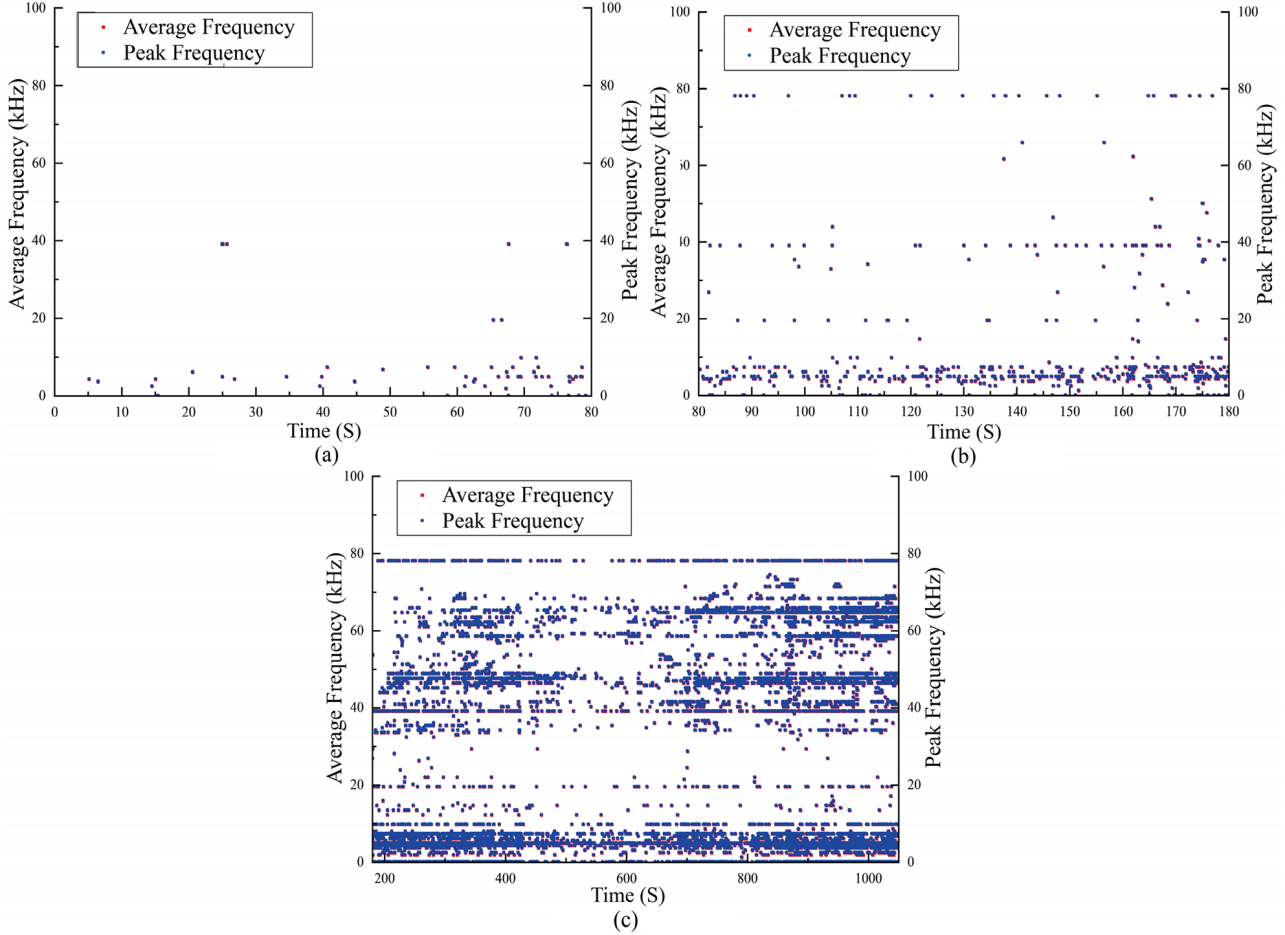
Frequency characteristics of AE signals at different stages. (**a**) Densification stage. (**b**) Shearing stage. (**c**) Failure stage.

This study also reveals the evolution law of frequency characteristics of AE waveform parameters in three stages with time, as shown in Fig. 11. It can be seen from the figure that in the compaction stage, the high-frequency signals are gradually less; in the shear stage, more high-frequency signals begin to appear, and the frequency signals of 50 ~ 100 kHz increase significantly; in the failure stage, many high-frequency signals appear, and the frequency signals of 50 ~ 100 kHz account for a large proportion. There are obvious differences in different stages, and the main difference is that there are more frequency signals in the failure stage, which can be used as an auxiliary criterion for the division of AE stages and the acquisition of precursory information of deformation and failure of loess.

## Discussion

### Validation of active waveguide model advantages

This study aims to verify previous findings by conducting a comparative test between the active and passive waveguide models of AE. It includes a comparative analysis of the characteristics of active and passive AE waveform parameters (energy, RDC, ASL, RMS, amplitude, peak frequency). This study confirms the effectiveness of the active waveguide model in monitoring data quality. The standardization of the active waveguide model monitoring improves the systematization and repeatability of monitoring and early warning systems for loess slope deformation and failure. Additionally, it mitigates the impact of different time, space, and external environmental conditions on AE signals. By generating AE signals through extrusion and shear friction of the filling material, this mechanism significantly reduces the influence of the surrounding geological environment on the AE monitoring data.

The stiffer the rock, the greater the energy storage limit and the more energy is released upon unloading. Due to the high stiffness property of quartz sand as a filling material, higher quality AE signals can be generated during extrusion and shear friction. Furthermore, according to the propagation law of elastic waves in bulk materials. Quartz sand has a faster elastic wave velocity due to its higher elastic modulus and lower Poisson's ratio, which can correspond to the RDC in acoustic emission signals. The high stiffness and thin wall thickness of the steel waveguide rods bring about a high AE signal with a low attenuation rate. The rubber tube used in the active waveguide model enhances its adaptability to various geological environments. The constitutes and advantages of the active waveguide system are shown in Table 1. The structure of the active waveguide model is flexible outside and rigid inside, which enhances the ability of the monitoring equipment to resist compression and shear. Therefore, the active waveguide model can not only monitor the deformation and failure of loess slope in real time and provide warning information, but also bear the structural deformation of loess and provide the function of support.

**Table 1:** AE constitutes and advantages of active waveguide system.

Constitutes	Description	Properties	Advantages	References
Filling material	Quartz sand	High angle, high stiffness, high elastic modulus, low Poisson ratio	Collisions produce higher energy and elastic wave velocities	Sun et al.^53^; Spriggs^54^; Zhang and Gao^55^; Smith et al.^25^
Waveguide	Steel waveguide	High stiffness, thin wall thickness	Collisions produce higher energy, low attenuation	Li et al.^56^; Wei et al.^57^; Zelenyak et al.^58^; Deng et al.^36^
Wrapping material	Rubber tube	Soft, thin wall thickness	High environmental adaptability, low attenuation	Heather-Smith et al.^59^; Deng et al.^34^; Deng et al.^35^

### Monitoring warning criterion

In view of the foregoing, the monitoring criterion of active waveguide system is established, and the deformation law of loess progressive failure is quantitatively described by using this technology. According to Fig. 5a,c, the deformation and cracking of the loess occur when signals with higher values start to appear. As more signals with higher values appear, the cracks in the loess expand, leading to collapse and spalling. Therefore, the sudden increase in AE energy and RDC is considered as a warning sign of loess slope deformation and failure. Similarly, in Figs. 9B and 10B, the loess starts to deform when the r-value and b-value change from a rapid increase to a rapid decrease. The collapse and spalling of the loess occur when the r-value and b-value change from a rapid decrease to a stable decrease. Therefore, while the stationary point of the AE r-value and b-value from sharp rise to sharp fall are considered as deformation precursors, while the inflexion of the AE r-value and b-value from rapid decline to stable decline is considered as a failure precursor. At present, a single criterion is insufficient to judge the occurrence of geohazards on loess slopes, and unpredictable external noise sources may appear at any time. Therefore, the combined application of the above criteria can provide a more accurate assessment of the development of geological disasters.

Currently, there is no specific AE signal processing method for loess. Most of the AE processing methods used are derived from rock AE technology and its extension technology. In this study, we investigate various kinds of AE signal processing techniques. These AE techniques are used to explore the monitoring application of active waveguide models in loess. We aim to uncover the evolution law of the AE signal in the active waveguide model. Additionally, we explore the failure mode and warning precursor information for monitoring loess slope deformation and failure using the active waveguide model. This study developed a valuable theoretical framework for the practical implementation of geological disaster prevention engineering.

**Figure 12:**
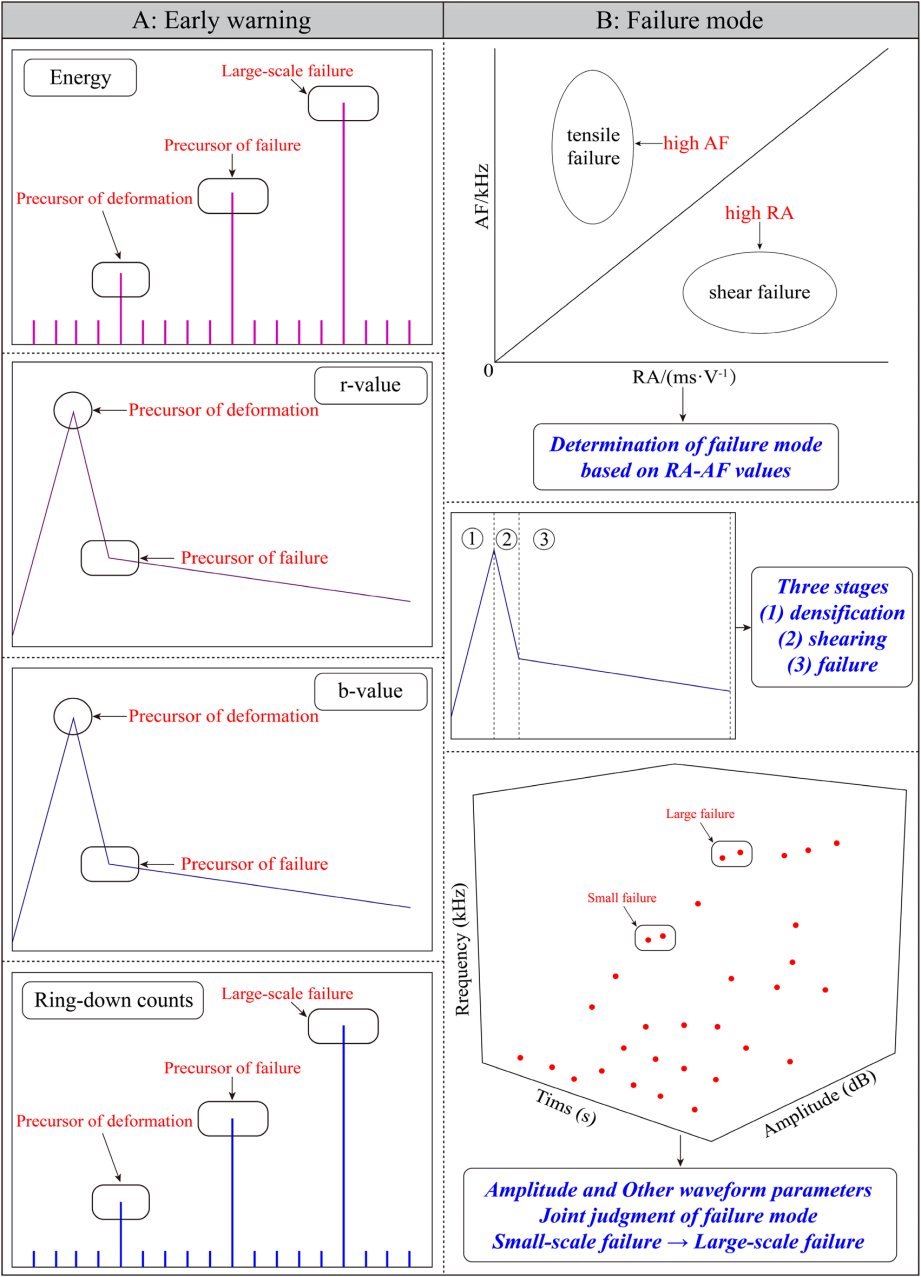
Active Waveguide System Monitoring Criterion. (**A**) Early warning characteristics. (**B**) Failure mode characteristics.

In this study, we explore the monitoring criterion system of the AE active waveguide system through a series of tests and analysis of the evolution law of the AE signal. This system includes two aspects: early warning characteristics and failure mode characteristics, as shown in Fig. 12. It can be used for acquiring and monitoring the precursor information of slope instability and failure in loess areas, as well as for prevention and early warning. The failure mode was identified based on the RA-AF value, and the change trend of AE frequency and amplitude was used to reflect the transition from small-scale failure to large-scale failure in loess. According to the characteristics of b-value and waveform parameters, the stages of loess deformation and failure are divided. The AE energy, RDC, and characteristics of the AE b-value and r-value were used as monitoring and warning precursor information.

**Figure 13:**
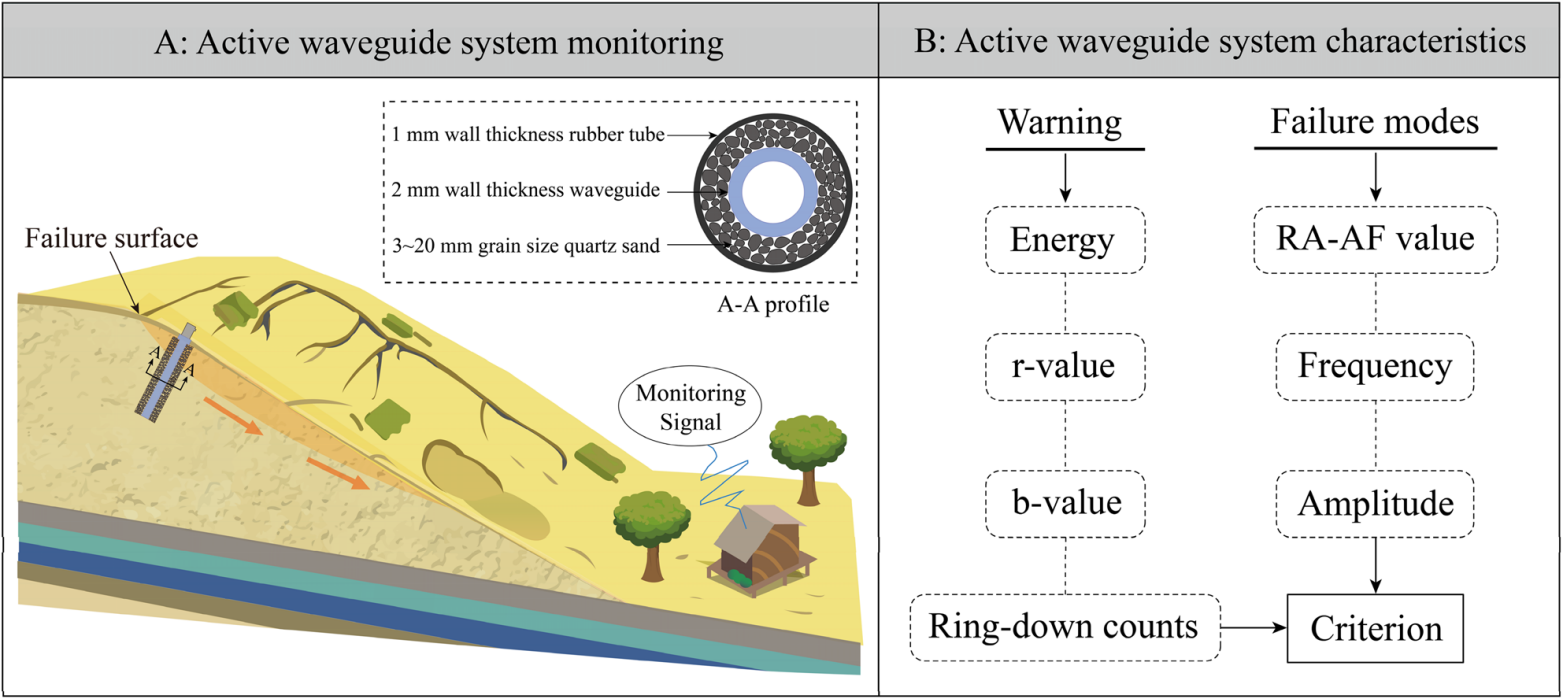
Schematic diagram of active waveguide model monitoring and characteristics. (**A**) Active waveguide system monitoring. (**B**) Active waveguide system characteristics.

Above all, in the field of geological hazard monitoring and prevention engineering, there are two main components: the monitoring system and the early warning criterion system, as shown in Fig. 13. The active waveguide monitoring system is made up of 3 ~ 20 mm quartz sand filling material, a 2 mm thick steel waveguide rod, and a 1 mm thick rubber tube. The early warning criterion for the active waveguide system consists of two parts, the failure mode, and the early warning criterion. The failure mode criterion includes the RA-AF value discrimination method, frequency characteristics, and amplitude characteristics. The early warning criterion includes AE energy, RDC, AE r-value, and b-value. The research on the early warning criterion of the active waveguide system presented in this paper is expected to have wide applications in the future for monitoring and early warning of geological disasters in loess areas.

## Conclusions

This paper presents compression loading tests conducted on active and passive waveguide models. The study focuses on analyzing the characteristics of AE waveform parameters in these models. Furthermore, the signal characteristics of active wave model monitoring loess deformation and failure test data are deeply analyzed. This analysis enhances the quality of AE monitoring data and explores the early warning criteria for the AE active waveguide system monitoring. The main conclusions are as follows:

1. The compression loading test of the active waveguide model and the passive waveguide model revealed that the AE waveform parameters (energy, RDC) of the active waveguide model were significantly better than those of the passive waveguide model. This confirms the data quality of the active guide model.
2. According to the signal characteristic discrimination method based on RA-AF value, it was determined that the RA value accounted for 74% in the active waveguide model, while the AF value accounted for 26%. This high RA value and low AF value indicate that the failure mode of the active waveguide model is shear failure. This finding provides an essential theoretical basis for identifying and acquiring information on loess deformation and failure precursors.
3. The characteristics of r-value, b-value, and frequency-amplitude are revealed when monitoring the deformation and failure of loess using the active wave-guide model. The deformation and failure of loess can be categorized into three stages: compaction stage, shear stage (elastic–plastic deformation stage), and failure stage, based on the distribution of b-value and the characteristics of AE waveform parameters.
4. This study explores the early warning criterion characteristics of the AE active waveguide system. The failure mode criterion is determined using the RA-AF value discrimination mode, as well as the frequency and amplitude characteristics. Additionally, the early warning criterion includes waveform parameter characteristics, as well as the AE r-value and b-value characteristics.

## Data Availability

The original contributions presented in the study are included in the article/Supplementary Material, further inquiries can be directed to the corresponding author.
